# Untangling connections between challenges in the adoption of learning analytics in higher education

**DOI:** 10.1007/s10639-022-11323-x

**Published:** 2022-10-20

**Authors:** Asma Shannan Alzahrani, Yi-Shan Tsai, Sehrish Iqbal, Pedro Manuel Moreno Marcos, Maren Scheffel, Hendrik Drachsler, Carlos Delgado Kloos, Naif Aljohani, Dragan Gasevic

**Affiliations:** 1 Centre for Learning Analytics, Faculty of Information Technology, Monash University, Clayton, Australia; 2 University Carlos III de Madrid, Getafe, Spain; 3 Ruhr-Universität Bochum, Bochum, Germany; 4 DIPF – Leibniz Institute for Research and Information in Education, Frankfurt, Germany; 5 King Abdul-Aziz University, Jeddah, Saudi Arabia

**Keywords:** Higher education, Learning analytics, Adoption challenges

## Abstract

Potential benefits of learning analytics (LA) for improving students’ performance, predicting students’ success, and enhancing teaching and learning practice have increasingly been recognized in higher education. However, the adoption of LA in higher education institutions (HEIs) to date remains sporadic and predominantly small in scale due to several socio-technical challenges. To better understand why HEIs struggle to scale LA adoption, it is needed to untangle adoption challenges and their related factors. This paper presents the findings of a study that sought to investigate the associations of adoption factors with challenges HEIs face in the adoption of LA and how these associations are compared among HEIs at different scopes of adoption. The study was based on a series of semi-structured interviews with senior managers in HEIs. The interview data were thematically analysed to identify the main challenges in LA adoption. The connections between challenges and other factors related to LA adoption were analysed using epistemic network analysis (ENA). From senior managers’ viewpoints, ethical issues of informed consent and resistance culture had the strongest links with challenges of learning analytic adoption in HEI; this was especially true for those institutions that had not adopted LA or who were in the initial phase of adoption (i.e., preparing for or partially implementing LA). By contrast, among HEIs that had fully adopted LA, the main challenges were found to be associated with centralized leadership, gaps in the analytic capabilities, external stakeholders, and evaluations of technology. Based on the results, we discuss implications for LA strategy that can be useful for institutions at various stages of LA adoption, from early stages of interest to the full adoption phase.

## Introduction

Learning analytics (LA) has been recognized widely as a promising field of research and practice that can offer opportunities to enhance learning, teaching, and institutional management (Tsai et al., 2020). The importance of LA is more prominent today than ever since higher education institutions (HEIs) transitioned rapidly towards online learning as a result of the COVID-19 pandemic outbreak (Adedoyin & Soykan, 2020). LA has shown promise in supporting adaptive learning (Johnson et al., 2016), improving students’ success (Rebecca Ferguson, 2012), tracking students’ performance (Gašević et al., 2015), identifying factors that improve student retention or predict attrition (Wong, 2017), and providing personalised feedback (Pardo et al., 2019). However, when HEIs start adopting LA, many challenges may arise, such as cultural and ethical issues, that can discourage stakeholders from using LA (Rebecca Ferguson, 2019; Macfadyen et al., 2014; Slade & Prinsloo, 2013).

Over the years, researchers in LA have sought to answer questions about the challenges of LA adoption and those faced by institutional leaders when adopting LA. For example, Tsai et al. (2020) identified prominent challenges and concerns related to stakeholder engagement and buy-in, weak pedagogical grounding, resource demand, and ethics and privacy. Several researchers have developed approaches to addressing challenges and concerns in these areas (Colvin et al., 2017; Sclater, 2016; Tsai et al., 2018). For example, a code of practice was developed that can be used as a taxonomy of ethical, legal, and logistical issues for LA (Sclater, 2016), While existing research in LA has made significant progress in documenting challenges that HEIs face (e.g., stakeholder buy-in, ethics, and privacy), researchers have also acknowledged that the challenges that confront LA are complex in nature and many are intertwined (Colvin et al., 2017; Macfadyen et al., 2014; Tsai et al., 2019). This paper thus seeks to untangle the connections between LA adoption challenges.

Tsai et al. (2021a) study was conducted to understand associations of different challenges with other related factors that influence LA adoption, and it is the first study that addressed these aspects in LA adoption. The study explored challenges by comparing LA experience among universities (novice and experienced HEIs). The results showed that both novice and experienced institutions demonstrated strong connections between challenges and ethics. However, novice institutions showed a particular challenge with data access when it comes to ethics and privacy issues. To date, there is limited research about the connections between different types of challenges that HEIs face in LA adoption and their related factors.

Moreover, there is little research exploring the differences in links between challenges that HEIs may face and scopes of LA adoption. The study's objective in this paper was to advance the understanding of factors associated with challenges in LA adoption across HEIs at different scopes of adoption (i.e., none, preparation-partial (prep-partial), and full). The objective of this study is also to identify changing patterns that may emerge from institutions with varying adoption scopes of LA, so as to provide insights that may help institutions to be prepared for challenges that they may face and better consider possible ways to overcome them in different LA adoption scopes whether none, prep-partial, or full adoption. We adopted quantitative ethnographic methods, including semi-structured interviews and epistemic network analysis (ENA). Our aim is to inform HEIs of their strategic adoption of LA. To this end, we were guided by this research question: **What factors are associated with the LA adoption challenges among institutions in different LA adoption scopes?**

## Literature review

### Learning analytics adoption

LA is rapidly gaining attention in online and technology-enhanced education (TEL) (Khalil & Ebner, 2015a; Lee et al., 2020). The literature indicates that although interest in LA is high, its adoption is still immature among HEIs (Tsai & Gasevic, 2017; Viberg et al., 2018). Different kinds of models have been developed to provide guidance to adopt LA in HEIs. For example, Greller and Drachsler (2012) proposed a framework that recognizes stakeholders' perceptions, expectations, and levels of understanding as key dimensions in LA, while ethics and privacy are identified as the main constraints for using LA. To support the adoption of LA, the Open University in the UK proposed the “Policy on Ethical use of Student Data for Learning Analytics” (The Open University, 2015). However, Tsai et al. (2018) and Tsai and Gasevic (2017) suggest that general guidelines for privacy, ethics, and other relevant factors may not always apply to the unique context of each institution. In light of this, Tsai et al. (2018) developed the SHEILA framework to guide HEIs throughout creating a full policy and strategy that addresses the needs of their specific contexts and stakeholders. The SHEILA framework was developed through multi-stakeholder involvement (i.e., students, teaching staff, senior managers, and policy-makers) to document common action points, policy questions, and challenges HEI may face. While SHEILA offers a rich foundation for HEIs to inform their LA strategy and policy, there is limited understanding in the literature about how challenges are mutually connected or related to other factors that shape LA adoption. This is precisely the gap in the literature that the study reported in this paper addresses.

### Challenges in learning analytics adoption

Challenges in LA adoption are essentially rooted in areas including technical factors, human factors, and organizational factors. Siemens (2013) posits that institutions can successfully adopt LA by addressing the challenges related to leadership, institutional culture, data access and security, technological infrastructure, ethical dilemmas, and the required learning skills. Other authors indicate that challenges in LA adoption can be in a lack of institutional capacity, funding, and infrastructure (Ngqulu, 2018), insufficient resources, and skills and expertise (Tsai et al., 2019). In addition, Tsai and Gasevic (2017) identify several challenges related to strategic planning and policy for LA adoption, such as a shortage of academic leadership, insufficient training of relevant stakeholders, and a lacuna of studies empirically validating the impact of LA. Existing research has also identified several factors that challenge LA adoption. Among these factors, stakeholders’ involvement has been widely cited (Dollinger & Lodge, 2018; Tsai & Gasevic, 2017), in addition to ethics and privacy (Kitto & Knight, 2019; Tsai et al., 2020). While adoption challenges have been studied broadly, few studies have investigated challenges in LA adoption of HEIs on different scopes of adoption (e.g., none vs. preparing or partially adopted vs. fully adopted).

Based on the LA adoption literature, we may conclude that common challenge-related factors are: ethics, stakeholders’ involvement, leadership, analytic culture, analytic capabilities, and technological infrastructure. These six factors were selected because they appeared to be the most challenging factors in the field of LA adoption (for more details about including these factors, see Section 3). These factors are discussed further in the remainder of this section and explored in the current study.

#### Ethics

In recent years, ethics has attracted significant attention in the field of LA (R. Ferguson et al., 2016; Slade & Prinsloo, 2013), including several proposals specifically designed to address ethical issues in HEIs (Drachsler & Greller, 2016; Kitto & Knight, 2019; Pardo & Siemens, 2014; Sclater, 2016). In their proposed checklist that aimed to inform decision-making in LA, Drachsler and Greller (2016, p. 90) define ethics as “a moral code of norms and conventions that exists in society externally to a person”. With the increasing use of student’s data, ethical concerns have emerged as a series of challenges that need to be addressed (Siemens, 2013). Studies that engaged with various stakeholders have also shown that the majority of the participants consider ethics and privacy to be the most important challenge to address (Drachsler & Greller, 2012; Hilliger et al., 2020; Jones, 2019; Kollom et al., 2021; Whitelock-Wainwright et al., 2019). Ethical challenges can be related to the duty to act, informed consent, safeguarding, equality and justice, data ownership and protection, and privacy and integrity of self (Rebecca Ferguson, 2019).

#### Leadership

Leadership has become increasingly important for the maturity of LA in HEIs (Tsai & Gasevic, 2017). Two approaches to leadership (top-down and bottom-up) in the adoption of LA in HEIs have been identified by (Dawson et al., 2018) based on interviews with senior managers at Australian universities. These two approaches have different strategies. A top-down approach focuses on LA infrastructure more than staff capacity. Thus, it can be difficult to gain recognition and acceptance among relevant stakeholders (Colvin et al., 2017), whereas a bottom-up approach focuses on consultation to improve staff awareness about the use of LA in higher education. However, bottom-up approaches are associated with the shortage of strategies that foster LA practices across an HEI (Dawson et al., 2018). The lack of support from senior management in bottom-up approaches can slow down the LA adoption progress (Tsai et al., 2019). Thus, top management support plays a significant role in the process of implementation of LA in HEIs.

#### Analytics culture

Analytic culture is an important factor in LA adoption (Tsai et al., 2020) and LA readiness (Arnold et al., 2014a). The importance of analytic culture is highlighted by the fact that stakeholders’ attitudes toward LA can vary significantly across HEIs (Hilliger et al., 2020; Kollom et al., 2021; Whitelock-Wainwright et al., 2020). Culture in LA can be analysed differently, whether as a culture of institutions accepting or using data to inform decision making (Oster et al., 2016) or as staff culture to engage in conversations around the results provided by LA (Dawson et al., 2018). Thus, the lack of analytic culture can be a challenge in LA adoption, and it is vital to understand how the culture can influence stakeholders’ intention to use LA and how different stakeholders accept, change, and move forward with the change that LA adoption may bring.

#### Analytics capabilities 

Expertise required for data analytics involves extracting valuable information from educational data and determining which data is more beneficial to achieving organizational goals within a time frame (Tulasi, 2013). Without appropriate data analytic expertise, HEIs can find it difficult to take advantage of data (Tsai et al., 2019).

#### Stakeholders involvement 

Typical stakeholders involved in LA in HEIs include students, teaching staff, institutional management, researchers, and developers (Drachsler & Greller, 2012; Khalil & Ebner, 2015b; Tsai et al., 2018, 2021b). Stakeholders can be divided into clients and subjects (Drachsler & Greller, 2012; Kollom et al., 2021). Data clients are users of LA who are qualified and expected to respond to the results of LA (e.g., teachers). Data subjects are data providers (e.g., students) (Drachsler & Greller, 2012; Kollom et al., 2021). The lack of stakeholders’ buy-in has been identified as a challenge that needs to be addressed in order to achieve successful adoption of LA (Greller & Drachsler, 2012; Lester et al., 2017; Tsai & Gasevic, 2017; Tsai et al., 2019, 2020). Stakeholder involvement has important implications for LA adoption, such as meeting stakeholders’ expectations and needs and ensuring responsible adoption (Knight et al., 2016; Whitelock-Wainwright et al., 2019, 2021).

#### Technology 

Technology infrastructure has been considered as one of the critical factors for building the capacity of HEIs for LA (Norris & Baer, 2013) and as “foundation elements” in LA implementation (Arnold et al., 2014c). Educational data can be collected, stored, processed, managed, and viewed using technology infrastructure, including analytic tools and applications (Macfadyen et al., 2014). The lack of appropriate infrastructure can negatively impact LA's deployment (Arnold et al., 2014b).

To sum up, the current study aimed to understand how all six factors (ethics, leadership, analytical culture, analytical capabilities, stakeholder involvement, and technology) are associated with challenges in adopting LA.

## Methods

The current study aimed to investigate the associations of challenges of LA adoption and their related factors in HEIs and compare HEIs with different scopes based on senior managers’ perspectives. To meet these aims, we carried out 52 interviews with 65 senior managers from 44 European HEIs from August 2016 and February 2017. The majority of the interviews were conducted in the UK (*n* = 34) and Spain (*n* = 14) due to the availability of institutional leaders. The participants in the interviews ranged from (Vice) Principals/Deans of Learning and Teaching to Heads of IT, Directors of E-learning Centres, and positions established especially for LA research and development. A total of 10 interview questions were developed to investigate (1) institutional plans for LA, (2) motivations for LA, (3) adopted strategy, (4) strategy development processes, (5) readiness preparations, (6) success and evaluation, (7) success enablers, (8) challenges, (9) ethical and privacy considerations, and (10) the interviewee’s views of essential elements in a LA policy. The questions were designed based on a literature review on key factors of LA adoption, which highlights context, strategy, people, and challenges (Tsai et al., 2021a). The interviews were semi-structured to allow the interviewer to adjust the order of questions or omit questions according to their assessment of what was most relevant to obtaining information concerning LA adoption (full interview questions can be found at http://bit.ly/leverage_interview). An opportunistic sampling (Tracy, 2013) method was adopted because access to the population was easy and inexpensive, with the additional benefit of the researchers’ existing network and influence. This is also a valid and advantageous method in qualitative research when the purpose is to understand the richness of a phenomenon rather than to generalise the findings. The institutions involved in this study differed in location, size, subject coverage, ranking, and LA adoption scopes. For further information about the sample, see https://bit.ly/Study_sample.

In order to analyse the interview data, all the interviews were transcribed and coded with a pre-defined coding scheme. In the first instance, a literature review (e.g., (Tsai & Gasevic, 2017), (Colvin et al., 2016))was used to develop the coding scheme, which evolved over time in an iterative process of reading and rereading the transcripts of interviews (Creswell & Poth, 2016), also described as a spiral process. The coding scheme was developed in an iterative process, applying both deductive and inductive methods. The final coding scheme contained 21 thematic groups under two types of variables – implementations and readiness. To ensure coding consistency, the principal researcher shared and explained the initial coding scheme to the other three researchers. Then, the four researchers coded two interviews independently to ensure consistency and resolve a disagreement. They repeated this until the inter-reliability of coding indicated a high level of agreement of over 85% based on the coding comparison query that was repeated twice with two different interviews. The final coding scheme contained 21 thematic groups under two types of variables – implementations and readiness. Each of these thematic groups contained 2 to 11 codes (for further information about the thematic codes, see https://bit.ly/LA_Thematic_group). For the scope of this paper, the investigation included seve thematic groups, including *challenges* (see Table 1) and the six challenge factors identified in the literature: ethics, stakeholders’ involvement, leadership, analytic culture, analytic capabilities, and technological infrastructure (see Sect. 2.2) The study focused on participants’ overview of the past, current, and potential challenges at their institutions or their perceptions about the negative impacts of LA. In our analysis, we focused on identifying connections between the six factors and their sub-codes (they can be found at https://bit.ly/Codes_and_subcodes.) and the seven sub-codes within the theme of challenges in LA adoption.

**Table 1:** Challenges sub-codes and descriptions

Abbr	Code	Description
C.EaP	Ethics and Privacy	Ethics and privacy related challenges (e.g., lack of unified consent system, accessibility of data, and data exchange protocols with external partners)
C.Cap	Capabilities	Challenges associated with institutional capabilities (e.g., lack of knowledge and skills to interpret data)
C.DL	Data limitation	Challenges related to ‘what data can do’ (e.g., quality of data and limitations in providing a complete picture about learning or learners)
C.R	Resource	Resource related challenges (e.g., technological infrastructure, human resources, and funding)
C.BIn	Stakeholder buy-in	challenges related to buy-in, including stakeholder attitudes towards LA (e.g., stakeholders at different levels hold different concerns)
C.M	Methodology	Methodology related challenges (e.g., not considering the context, and failing to recognise that data cannot provide a complete picture of learning),
C.Rel	Relevance	Challenges pertaining to relevance (e.g., the usefulness of LA in addressing institutional problems and goals)

As a part of the analysis of the content, we labelled institutions according to their adoption scope in order to find out whether associations between adoption factors varied among institutions with different adoption scopes: (1) *none* (*n* = 19) – Institutions expressed interest in LA, but have not taken action to prepare for LA activities, 2) *preparation-partial (prep-partial)* (*n* = 14) – Institutions had taken action in preparation for LA development or had implemented LA on a small scale, such as a pilot study, and 3) *full* (*n* = 11) – Institutions had implemented LA in an institution-wide scale. Note that only four institutions had been preparing to adopt LA. Due to the relatively small sample size and similarities observed in our qualitative analysis of the challenges reported in the interviews, this group was merged with the partial adoption group in our analysis.

The coded interview transcripts were analysed using epistemic network analysis (ENA). ENA is “a collection of techniques for identifying and measuring the connections between elements in coded data and representing them in dynamic network models” (Shaffer et al., 2016, p. 9). ENA was particularly suitable to address the study aims as it allows for the analysis of co-occurrence of codes (i.e., epistemic networks), visualization of the structure of epistemic networks, and statistical comparison of epistemic networks of different groups (Csanadi et al., 2018). ENA is developed purposefully to handle challenges involving a limited collection of codes and modeling the structure of connections between codes with highly dynamic and dense interactions. Singular value decomposition (SVD) is used to transform a high-dimensional network of connections between codes into low-dimensional space, guaranteeing that units of analysis with similar patterns are closer together and those with different patterns are further apart.

ENA models have three fundamental components: *units of analysis*, *conversations* or *stanzas*, and *codes*. Units of analysis identify the components of the data for which the model will build epistemic networks; conversations or stanzas identify the boundaries within which the model identifies connections; and codes identify the concepts which the model identifies connections between. For a given unit of analysis, ENA identifies connections between codes if those codes co-occur in the same conversation or stanza. In this study, we adopted ENA, which has been used to successfully identify connections between adoption factors of LA based-on interviews (Tsai et al., 2021a). We selected individual institutions as our unit of analysis, two consecutive conversation utterances in our interviews were the conversations (or stanzas), and sub-codes for challenges and other factors were used as codes. Because we were interested in the associations *between* these two sets of codes (i.e., sub-codes under challenges and sub-codes under other factors), and not the associations *within* them, we applied a mask to our ENA model such that the only connections present in the model were those between the two sets of codes (i.e., connections between sub-codes under challenges and those under other factors). We used mean rotation along the X-axis to visualise the differences between groups. We also provided the t-test results of the network means on the X-axis, the subtraction plots, and the subtraction networks (as included in the appendices). Note that due to the nature of epistemic network analysis and the requirement to apply mean rotation for each pair of groups that are compared, it was not possible to perform ANOVA. The connections between each investigated code were additionally triangulated with relevant quotes to illustrate the connections. To distinguish the 44 institutions, the interview quotes are designated with a ‘U’ and a number between 1 and 44.

## Result

### Challenges and ethics

The *Ethics* codes considered the extent to which ethical considerations have been taken into account by a HEI. There are seven sub-codes under this theme (Table 2):

**Table 2:** Ethics sub-codes and descriptions

Abbr	Code	Description
E.LAw	Limited awareness	There was low to no awareness of ethical implications of LA
E.LD	Limited discussion	Participants were aware of certain ethical issues but did not consider them as pertinent to the current state of adoption
E.T	Transparency	In the design and implementation of LA, transparency was given significant consideration (e.g., being clear about what data was collected, how data was used, collected and stored)
E.C	Consent	In the design and implementation of LA, consent-seeking was carefully examined
E.Ano	Anonymity	Consideration of anonymity principles
E.Ac	Access	Consideration of access to data
E.Ow	Ownership	Consideration of the ownership of data

**Fig. 1:**
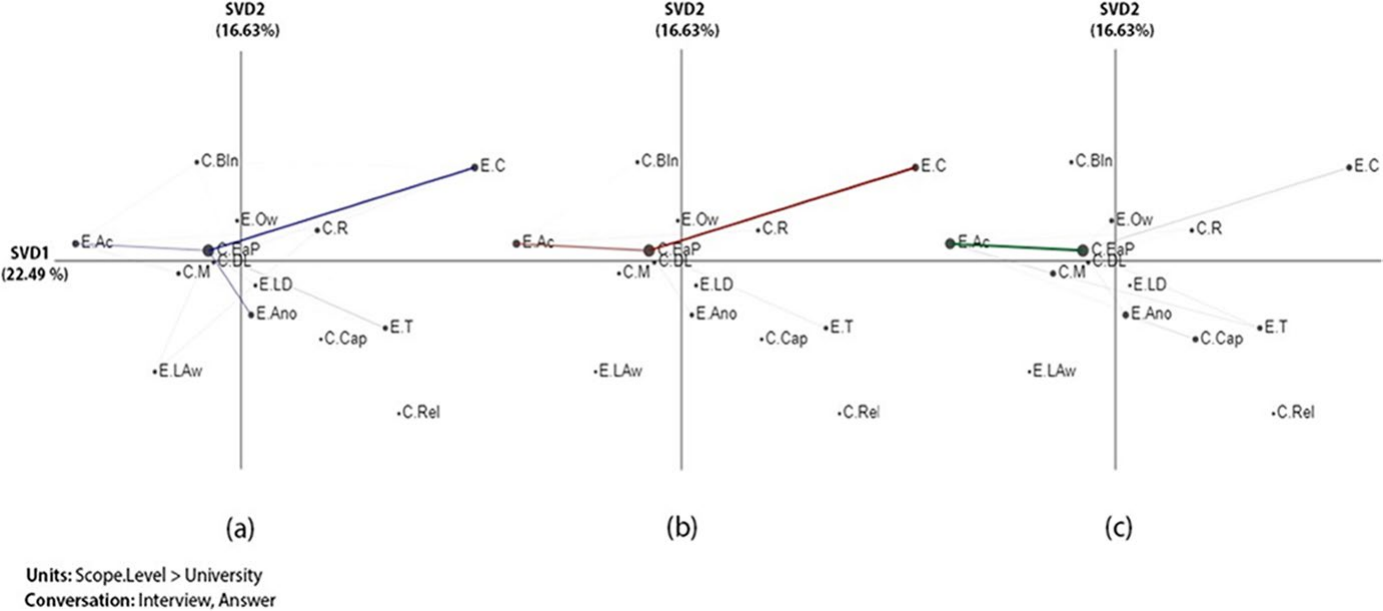
Associations of challenges and ethics: (a) none, (b) prep-partial, and (c) full adoption institutions

The x-axis (SVD1) explained 22.49% of the variance in the networks of the institutions, while the y-axis (SVD2) explained an additional 16.63% of the variance in the networks that analysed links between challenges and ethics (Fig. 1). An independent two-sample t-test showed significant differences along the X-axis between *n*one and *p*re-partial (t (25.486) = -3.3069, *p* = 0.003), between *none* and *full* (t (15.48) = -3.43, *p* = 0.004), and between *p*re-partial and full (t (21.94) = -2.50, *p* = 0.021). For more information about statistical results among each two groups (none-prep-partial, None-full, and perp-partial- full) (see Table 8 in Appendix 1), the projection of the centroids of individual institution networks ( see Fig. 7 in Appendix 2), and the subtracted networks (see Fig. 8 in Appendix 2).

ENA revealed that all institutions, regardless of LA adoption scopes, faced challenges related to ethics and privacy. The results showed that both none- and prep-partial adoption institutions had particularly strong connections of challenges related to ethics and privacy (*C.EaP*) with the ethical factor of data consent (*E.C*) (Fig. 1 (a) and (b)). This indicates that consent seeking was the most prominent challenge related to ethics and privacy before institutions reached the institution-wide adoption of LA. For example, one interviewee from U36 (none adoption) indicated:

I think that there is a sort of rush to deliver something that other institutions are seen to be delivering for their students. And I think sometimes the nuances of the difficulties and the concerns especially around getting proper informed student consent and creating algorithms that reflect and don’t distort student activity. That kind of detail is lost a bit in the idea that actually we could, you know, we could create this, we could leverage these great big data sets in a way to really drive forward the student experience. *-U36*

The results also showed that both prep-partial and full LA adoption institutions had a strong connection between the challenge of ethics and privacy (*C.EaP*) and the ethical factor of access (*E.Ac*). This indicates that access to data was an issue that the institutions wrestled with once LA was implemented. This is particularly notable among the full adoption institutions compared to other ethics factors (Fig. 1(c)). For example, one interviewee from U40 (full adoption) explained this challenge:

There are some challenges here with the exchange of data. So, the students’ union would like to have much more access to this system but they can’t because they are a separate entity. So, it’s about being clear about who has access to data. *-U40*

### Challenges and leadership

Leadership in our study had three sub-codes (Table 3):

**Table 3:** Leadership sub-codes and descriptions

Abbr	Name	Description
L.Dec	Decentralized	There was little or no evidence of centralised leadership (i.e., LA is a grassroot activity and is not supported by the central university management)
L.C	Centralized	LA is driven by top-down leadership from a limited number of sources (e.g., led by the vice chancellor or leaders in the Teaching and Learning Unit)
L.Dis	Distributed	Is a form of shared leadership that creates a breeding ground for new leaders with resources and structures that allow them to lead in their areas of influence

**Fig. 2:**
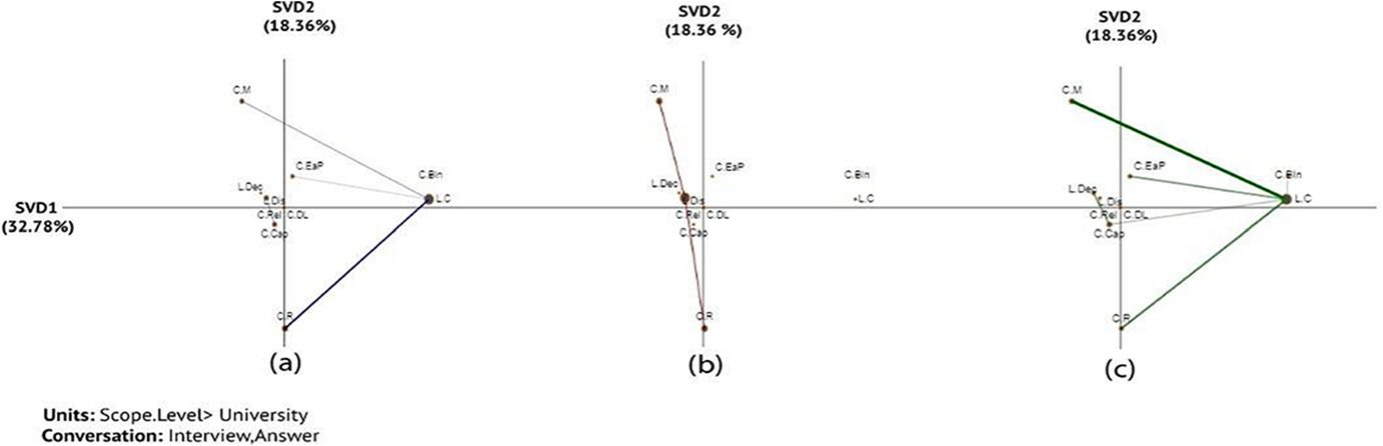
Associations of challenges and leadership: (a) none, (b) prep-partial, and (c) full adoption institution

The x-axis (SVD1) explained 32.87% of the variance in the networks of the institutions, while the y-axis (SVD2) explained an additional 18.36% of the variance in the networks that analysed links between challenges and leadership (Fig. 2). An independent two-sample t-test showed significant differences along the X-axis between none and pre-partial (t (30.40) = -2.22, *p* = 0.034) and between *pre-partial and full* (t (11.17) = -2.45, *p* = 0.032). However, there was no significant difference along the X-axis between none and full (t (13.43) = -1.81, *p* = 0.093). For more information about statistical information among each two groups (none-prep-partial, None-full, and perp-partial- full) (see Table 8 in Appendix 1), the projection of the centroids of individual institution networks (see Fig. 9 in Appendix 3), the subtracted network (see Fig. 10 in Appendix 3).

The results of ENA showed different connections across institutions with different LA adoption scopes. Centralised leadership (*L.C*) was found to be most prominent among the none and full adoption institutions (Fig. 2(a, c)), whereas distributed leadership was particularly present among the prep-partial adoption institutions (Fig. 2(b)) partly due to the fact that partial implementation including pilots of learning analytics tended to be led by leaders in a department level or by individual staff members (see Sect. 3 for ‘scale’ definitions). When inspecting connections between the leadership factor and challenges, methodology (*C.M*) and resource (*C.R*) (Fig. 2) challenges emerged regardless of leadership types (Fig. 2). However, when comparing the non-adoption and full-adoption HEIs with the same prominence of centralised leadership (*L.C*), there was a particularly strong connection with resource challenges (*C.R*) for the none-adoption and with the methodology challenges (*C.M*) for the full-adoption HEIs. This implies that institutions new to LA struggled more with resources, but those that had implemented LA fully struggled more with getting the adoption methodology right despite having a strong presence of centralised leadership. For example, one interviewee from U22 (none adoption) explained various challenges, including the lack of people with expertise in LA and funding:

And the staff expertise.... A lot of the support services in the universities have really been stretched in terms of the resources – understaffing. So the numbers of staff has gone down, the budgets have gone down. And I think that is a real challenge actually to work on a major new project. And we’d really need to make sure that it’s properly resourced and that people are clear about what the level of investment is needed at the beginning, both in terms of software solutions but also in terms of appropriate expertise. *-U07*

Compared to the non-adoption institutions, the prep-partial institutions had more prominent presence of the methodology challenge (*C.M*) while their struggle of resources (*C.R*) continued (Fig. 2(b)). The connections of these two challenges with the distributed leadership (*L.Dis*) presence indicate that staff members that led LA pilot or small-scale adoption may have particularly wrestled with these challenges. It also shows growing awareness of ‘getting the adoption methods right’ once institutions move beyond the ‘interest’ stage (none adoption) to ‘action’ stage (prep-partial and full adoption). The challenge of implementation methodology continues to be evident in the full-adoption institutions though with a stronger presence of centralised leadership (*L.C*) (Fig. 2 (c)). For example, the interviewee from U26 (full-adoption) faced challenges related to data collection that did not reflect actual learning, resulting in risks of misleading decisions.

In fact, in learning itself is a process that...we could observe and infer from behaviours but quite difficult to actually collect data evidence on actual learning. So there’s a danger that in an environment and a context when metrics measures KPI’s are becoming increasingly important and, policy decisions like the introduction of the teaching excellence framework [...] So the Government in England at least have wanted to bring in this teaching excellence framework and wanted to do it quickly. And HEFCE have essentially been left in a position where they can only use the metrics that are available even if they are not the right metrics. *-U26*

### Challenges and analytics culture

The *Analytic culture* relates to the educational analytics culture at the institution and to any strategic initiatives to increase analytics culture. Analytics culture included the following sub-codes (Table 4):

**Table 4:** Analytical culture sub-codes and descriptions

Abbr	Name	Description
ACu.I	Immature	HEI in general had limited knowledge of LA
ACu.S	Strategic	There were endeavours to strengthen the institutional culture strategically by delivering workshops or addressing current cultural impediments to the implementation of LA
ACu.Ding	Developing	In general, higher education institutions expressed willingness in using LA to improve teaching practises and promote student learning
ACu.R	Resistant	The culture in which some stakeholders were adamantly opposed to LA for a variety of reasons (e.g., ethical concerns)
ACu.Dev	Developed	HEI in general had fairly good understanding of LA and acknowledge the potential of LA

**Fig. 3:**
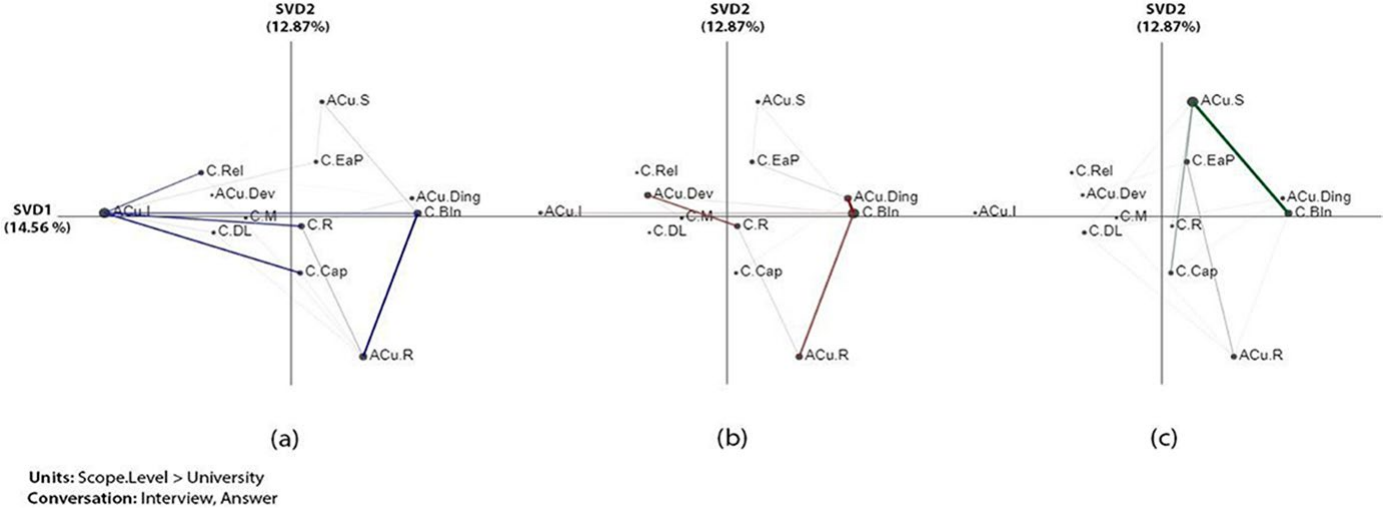
Associations of challenges and analytic culture: (a) none, (b) prep-partial, and (c) full adoption institution

The x (SVD1) and y axes (SVD2) explained 14.56% and 12.87% of the variance, respectively in the epistemic networks that looked at associations between challenges and analytic culture (Fig. 3). An independent two-sample t-test showed significant differences along the X-axis between n*one* and *pre-partial* (t (30.99) = -4.82, *p* = 0.001), between *none* and f*ull* ( t (25.07) = -6.04, *p* = 0.001), and between *pre-partial and full* (t (16.22) = -4.39, *p* = 0.001). For more information about statistical information among each two groups (none-prep-partial, None-full, and perp-partial- full) (see Table 8 in Appendix 1), the projection of the centroids of individual institution networks (see Fig. 11 in Appendix 4), and subtracted network (see Fig. 12 in Appendix 4).ana

The results showed that the challenge of stakeholders’ buy-in (*C.BIn*) had the most connections with the analytic culture factors in the institutions across all LA adoption scopes. There are also prominent connections between immature culture, developing culture, and developed culture with other challenge codes in the none and prep-partial groups. The ENA shows that the none adoption group demonstrated a strong connection of immature culture with several challenges, including the relevance of LA to the institution’s priorities, resources, and capabilities (knowledge skills). By comparison, the prep-partial group shows the emergence of both developing and developed cultures among different institutions in this group, and they’re especially connected to buy-in and resource challenges, respectively. The full-adoption institutions, however, do not demonstrate prominent connections between challenges and any of the three levels of cultural maturity.

The results also showed that both none and prep-partial adoption institutions demonstrated strong connections between the stakeholder buy-in (*C.BIn*) challenge and the resistance culture (*ACu.R*) (Fig. 3(a) and (b)). This indicates that a culture of resistance to LA is the most prominent factor of the buy-in challenge among institutions that have not yet reached institution-wide adoption. For example, one of the interviewees from U25 explained staff resistance due to workload.

But I do think there’s an element where there’s gonna be, ‘we want you to do this and we want you to implement learning analytics, and this will streamline the way that you evaluate your teaching practice, but to do this you’re gonna have to change the way that you teach’. And I think the people are then gonna go, ‘well that is gonna cut into my research time’. And that’s where the trade off’s gonna be. *-U25*

By contrast, full adoption institutions had a strong connection between stakeholders’ buy-in challenge and the strategic culture (*ACu.S*) (Fig. 3(c)), which shows that despite continuous struggles with stakeholder buy-in, institutions on a full adoption scale are more likely to have developed strategies to address the buy-in challenge in some ways. For example, an interviewee indicated:

There are a range of issues there in terms of staff development, but naturally we’ve been working with people who want to get involved, and using them as champions to try and convince or support other colleagues who are maybe a bit more reticent. *-U29*

### Challenge and analytics capabilities 

The *Analytic capabilities* code represented the evaluation of an institution’s analytic capabilities and improvements made to those capabilities. Analytical capabilities theme contained four sub-codes (Table 5):

**Table 5:** Analytical capabilities sub-codes and descriptions

Abbr	Name	Description
ACap.T	Training teachers	Training for teaching/support staff had been offered or was recognised as necessary
ACap.S	Training students	Training for students’ staff had been offered or was recognised as necessary
ACap.E	Experts	There were professionals assigned to help with data analysis and take on important LA-related activities
ACap.G	Gaps	There is recognition of gaps existing in the understanding of LA and skills for it among stakeholders at various levels

**Fig. 4:**
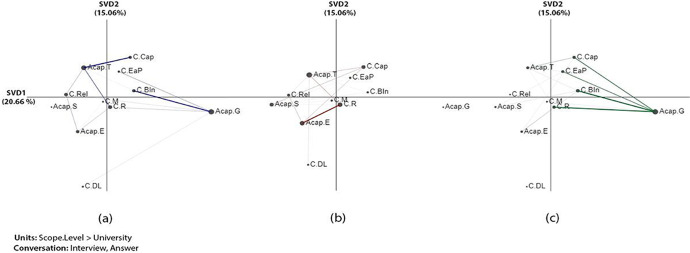
Associations of challenges and analytic capabilities: (a) none, (b) prep-partial, and (c) full adoption institution

The x (SVD1) and y (SVD2) axes explained the variance by 20.66% and 15.06%, respectively in the networks that looked at the associations between challenges and analytic capabilities (Fig. 4). An independent two-sample t-test showed significant differences along the X-axis between *none* and *pre-partial* (t (30.98) = -3.51, *p* = 0.001), between *none* and f*ull* (t (14.08) = -2.67, *p* = 0.018), and between *pre-partial and full* (t (10.62) = -3.20, *p* = 0.009). For more information about statistical information among each two groups ( none-prep-partial, None-full, and perp-partial- full) (see Table 8 in Appendix 1), the projection of the centroids of individual institution networks (see Fig. 13 in Appendix 5), and the subtracted network (see Fig. 14 in Appendix 5).a

The results of the ENA showed that the prep-partial adoption institutions demonstrated a strong connection between resourcing challenges (*C.R*) and experts in analytic capabilities (*ACap.E*), which indicates that institutions in this adoption scope still struggled with resources (e.g., infrastructure, funding, and human resources) despite the presence of LA expertise (Fig. 4 (b)). For example, an interviewee from U01 (prep-partial adoption) struggled to appoint an administrative lead for their LA working group due to ‘unclear ownership’ of LA despite having experts in LA:

We need someone who’s supporting, a project officer or a secretary for the group. And that is proving more difficult than I thought it would be. So our academic services seem to think that Information Services should support this activity. And Information Services seem to think academic services should support the activity. So there’s been a bit of back and forth around that. *-U01*

By contrast, both the none and full adoption institutions had strong connections between the stakeholders’ buy-in challenge (*C.BIn*) and gaps in the analytic capabilities (*Acap.G*), which indicates a certain level of relationship between stakeholder acceptance of LA and their understanding of LA regardless of the scope of adoption (Fig. 4 (a) and (c)). A participant from U29 (full adoption) explained their challenges with stakeholder buy-in and unequal understanding and analytical capabilities, which could be resulted from an entrenched culture:

This is not specific to learning analytics, and I don’t think it’s specific to U7 that there will be some colleagues who will find this more challenging than others, and may even not want to engage with the project. And I think some of that will be cultural, and some of it may be because they are naturally sceptical. *-U29*

In addition, the results showed that as institutions reached full adoption, they encountered a wider range of challenges in connection to the gaps in key stakeholders’ understanding of LA. In addition to buy-in, the connections also include resources (*C.R*), ethics and privacy (*C.EaP*), and capability (*C.Cap*) Fig. 4(c)).

### Challenges and stakeholder’s involvement

The *stakeholder involvement* theme identified the process by which HEIs communicated and consulted different stakeholders in the planning or implementation of LA. This was represented by six sub-codes (Table 6):

**Table 6:** Stakeholders involvement sub-codes and descriptions

Abbr	Name	Description
S.HL	High-level	Senior managers
S.SL	Support-level	IT units and administrative services
S.T	Primary-teachers	Teachers as primary stakeholders
S.S	Primary-students	students as primary stakeholders
S.E	External stakeholders	LA service providers or other external partners
S.L	Limited	There was little evidence of consultation with or involvement of any stakeholders

**Fig. 5:**
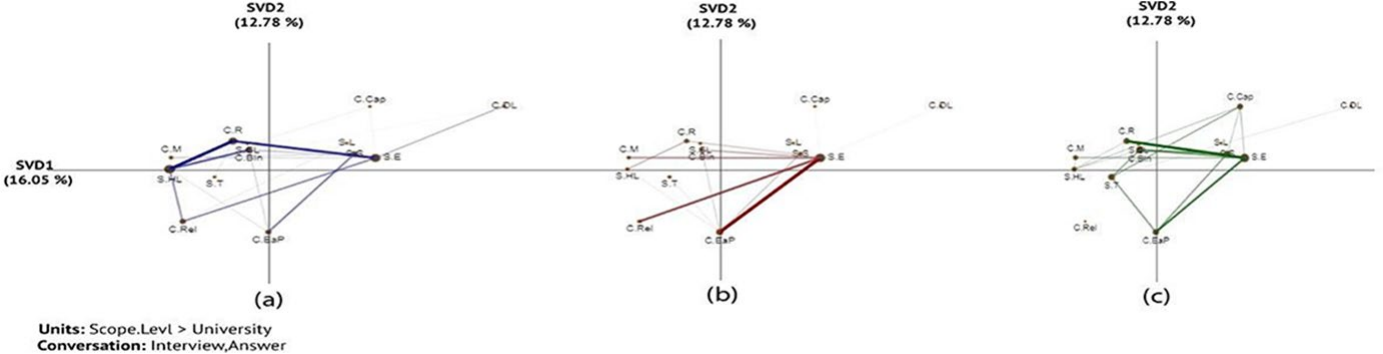
Associations of challenges and stakeholder involvement: (a) none, (b) prep-partial(c) full adoption institutions

The x (SVD1) and y (SVD2) axes explained variances 16.05% and 12.78%, respectively in the networks (Fig. 5). An independent two-sample t-test showed significant differences along the X-axis between *none* and *pre-partial* (t (24.16) = -3.80, *p* = 0.001), between *none* and *full* (t (25.75) = -4.59, *p* = 0.001), and between *pre-partial* and *full* (t (17.864) = -3.15, *p* = 0.005). For more information about statistical information among each two groups ( none-prep-partial, None-full, and perp-partial- full) (see Table 8 in Appendix 1), the projection of the centroids of individual institution networks (see Fig. 15 in Appendix 6), and the subtracted network (see Fig. 16 in Appendix 6).

The result of ENA showed that the none and full adoption institutions had strong connections between external stakeholders (*S.E*) and the challenge of resources (*C.R*). This indicates that both groups of institutions are likely to work with external stakeholders to tackle resource issues, such as collaborating with services provider or external partners (Fig. 5 (a) and (c))). For example, a participant from U11 (non-adoption) explained:

I think institutionally there should be a national collaboration around it because Analytics is so difficult that, you know it’s resource heavy.*-U11*

The results of ENA also showed that the prep-partial and full adoption institutions had strong connections of ethics and privacy challenges (*C.EaP*) with external stakeholders (*S.E*) (Fig. 5 (b) and (c)).This could mean that these institutions worked with external partners to resolve ethics and privacy issues (such as Jisc and their code of practice(Sclater, 2016)), or that institutions’ interactions with external stakeholders brought ethics & privacy issues into prominence. For example, one participant from U01 (prep-partial adoption) indicated that the lack of global law related to data protection raise concerns regarding the exchange of data with external stakeholders.

The project is a collaboration between different universities in different countries. [It] is a challenge in terms of the laws that may influence the restrictions on how you handle the data. *-U01*

## Challenges and technology 

The *Technology* theme identified actions that the HEIs took to prepare themselves to develop technological infrastructure for LA. Technology theme had four sub-codes (Table 7):

**Table 7:** Technology sub-codes and descriptions

Abbr	Name	Description
T.ExP	External partnership	External partnership such as forming a cooperation with service providers in LA
T.En	Technology enhancement	Institutions implemented or developed IT systems
T.Ev	Technology evaluation	The HEIs assessed the existing IT system that is required for LA implementation
T.Nt	No technology evaluation	There have been no assessments of the capabilities of existing IT systems and software required to implement LA, nor of the possibilities of various forms of data (Note that this code is not present in the ENA networks as no connection was identified)

**Fig. 6:**
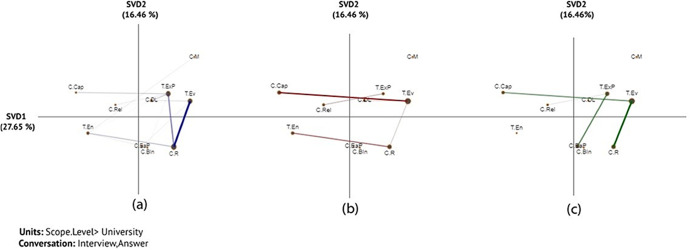
Associations of challenges and technology: (a) none, (b) prep-partial, and (c) full adoption institutions

**Fig. 7:**
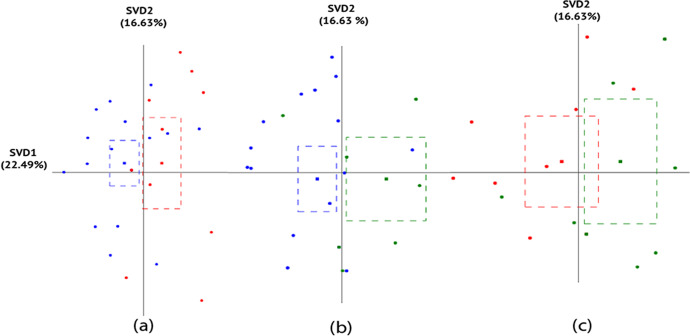
Blue dots are none LA adoption institutions, red dots are prep-partial LA adoption institutions, and green dots are full LA adoption institutions. The squares on the X-axis represent group means, and each square is surrounded by a rectangle representing 95% confidence intervals

**Fig. 8:**
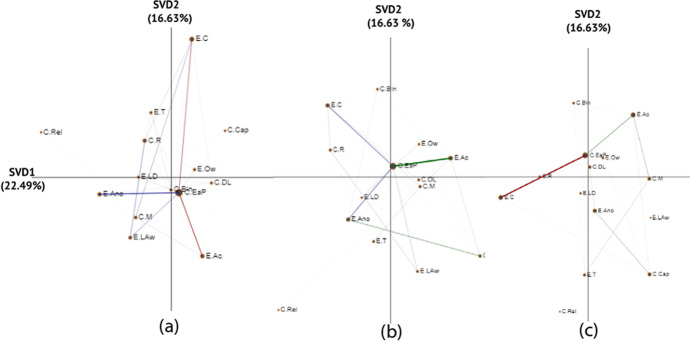
Subtracted network. In graph (a) subtracted network of none and prep-partial, (b) subtracted network of none and prep-partial, and (c) subtracted network of prep-partial and full

**Fig. 9:**
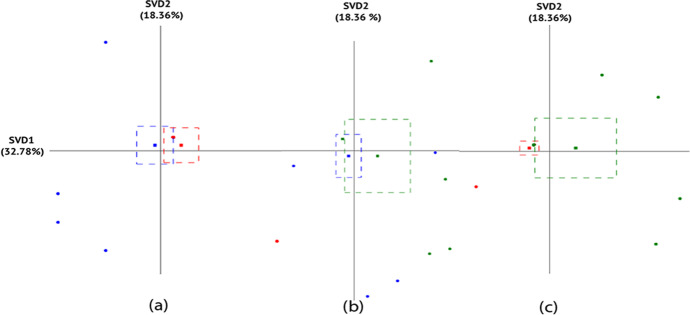
Blue dots are none LA adoption institutions, red dots are prep-partial LA adoption institutions, and green dots are full LA adoption institutions. The squares on the X-axis represent group means, and each square is surrounded by a rectangle representing 95% confidence intervals

**Fig. 10:**
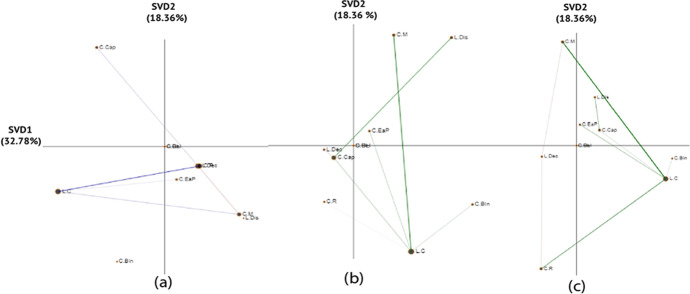
Subtracted network. In graph (a) subtracted network of none and prep-partial, (b) subtracted network of none and prep-partial, and (c) subtracted network of prep-partial and full

**Fig. 11:**
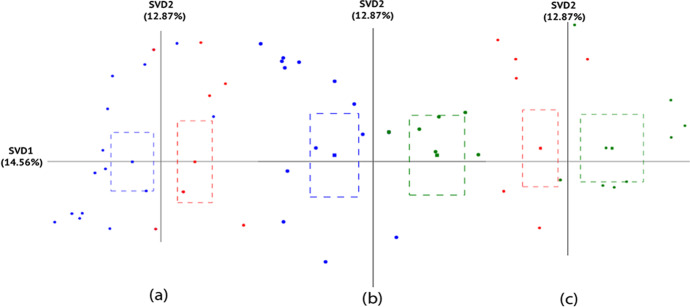
Blue dots are none LA adoption institutions, red dots are prep-partial LA adoption institutions, and green dots are full LA adoption institutions. The squares on the X-axis represent group means, and each square is surrounded by a rectangle representing 95% confidence intervals

**Fig. 12:**
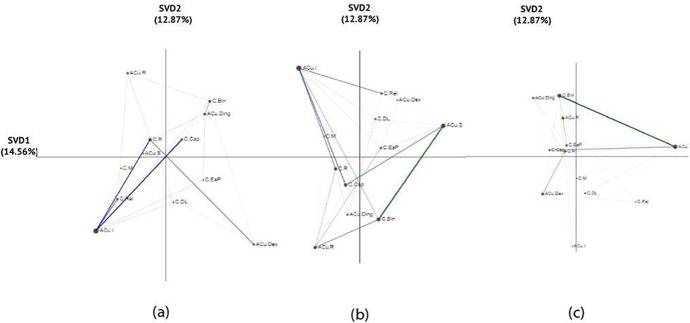
Subtracted network. In graph (a) subtracted network of none and prep-partial, (b) subtracted network of none and prep-partial, and (c) subtracted network of prep-partial and full

**Fig. 13:**
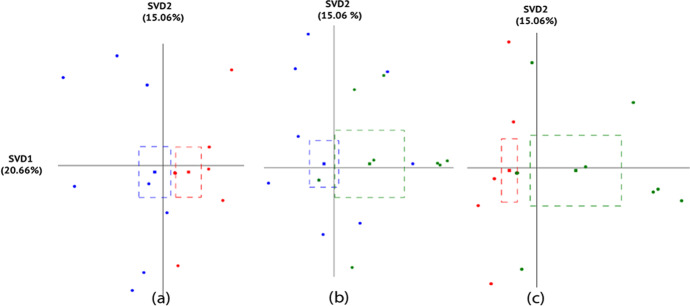
Blue dots are none LA adoption institutions, red dots are prep-partial LA adoption institutions, and green dots are full LA adoption institutions. The squares on the X-axis represent group means, and each square is surrounded by a rectangle representing 95% confidence intervals

**Fig. 14:**
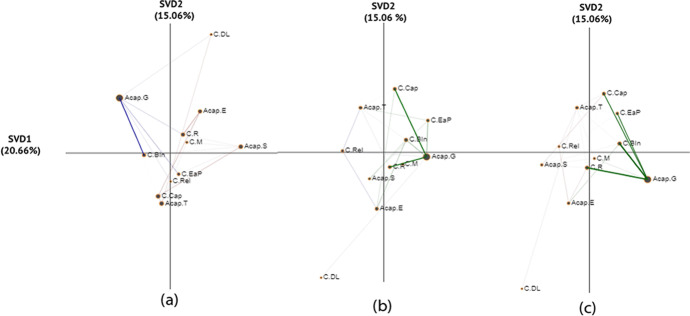
Subtracted network. In graph (a) subtracted network of none and prep-partial, (b) subtracted network of none and prep-partial, and (c) subtracted network of prep-partial and full

**Fig. 15:**
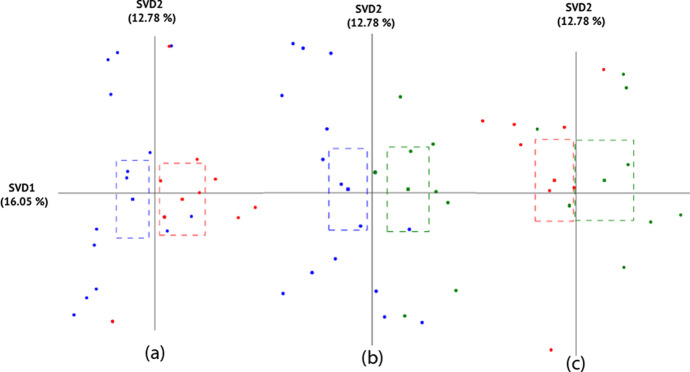
Blue dots are none LA adoption institutions, red dots are prep-partial LA adoption institutions, and green dots are full LA adoption institutions. The squares on the X-axis represent group means, and each square is surrounded by a rectangle representing 95% confidence intervals

**Fig. 16:**
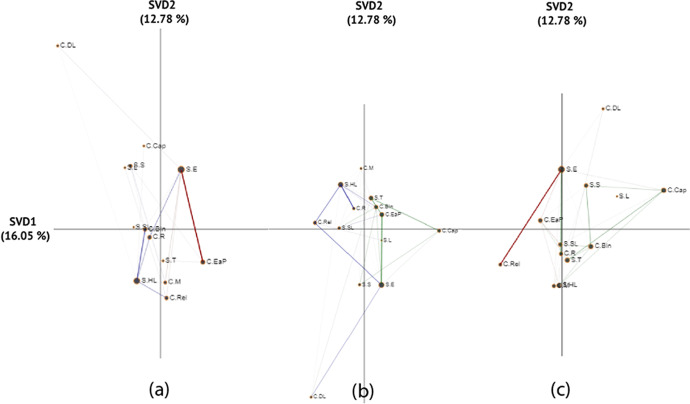
Subtracted network. In graph (a) subtracted network of none and prep-partial, (b) subtracted network of none and prep-partial, and (c) subtracted network of prep-partial and full

**Fig. 17:**
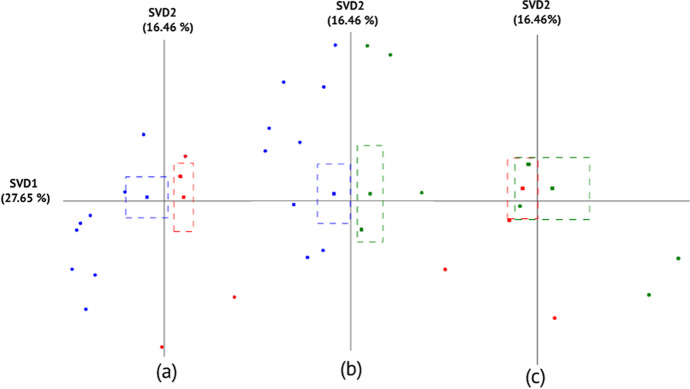
Blue dots are none LA adoption institutions, red dots are prep-partial LA adoption institutions, and green dots are full LA adoption institutions. The squares on the X-axis represent group means, and each square is surrounded by a rectangle representing 95% confidence intervals

**Fig. 18:**
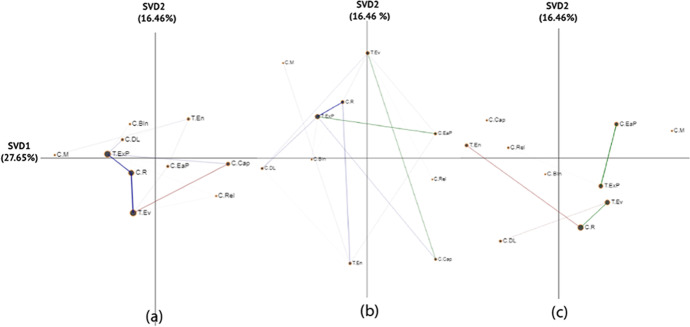
Subtracted network. In graph (a) subtracted network of none and prep-partial, (b) subtracted network of none and prep-partial, and (c) subtracted network of prep-partial and full

The x (SVD1) and y (SVD2) axes explained 27.65% and 16.65% of variance, respectively in the networks that analysed connections between challenges and technology (Fig. 6). An independent two-sample t-test showed significant differences along the X-axis between *none* and *pre-partial* (t (25.64) = -3.31, *p* = 0.003) and between *none* and *full* (t (28.99) = -3.71, *p* = 0.001). However, there was no significant difference along the X-axis between *pre-partial* and *full* (t (13.42) = -1.64, *p* = 0.125). For more information about statistical information among each two groups ( none-prep-partial, None-full, and perp-partial- full) (see Table 8 in Appendix 1), the projection of the centroids of individual institution networks (see Fig. 17 in Appendix 7), and the subtracted network ( see Fig. 18 in Appendix 7).

The results of ENA revealed that none and full adoption institutions had strong connections between the resourcing challenge (*C.R*) and evaluation of the technology (*T.Ev*) (Fig. 6 (a) and (c)). In line with the results presented earlier, resourcing challenges remained prominent even when institutions reached full adoption. For example, a participant from U31 (full adoption) indicated:

We’ve also been doing some work just to see whether or not, for example, we can take data from eduroam logins. And what that looks like when we analyse it. And how much trouble it’s going to be to analyse it. How much data is gonna be produced so therefore how much space you need and what sort of warehouse is required in order to accommodate those data. *U31*

Although the connection with the resourcing challenge is not as prominent among the prep-partial adoption institutions, the results showed a shift to capability challenges (*C.Cap*) (Fig. 6 (b)) in relation to technology evaluation (*T.Ev*). This indicates an emerging need of analytics expertise to carry out technology evaluation when institutions moved into the prep-partial adoption stage. This connection remains visible among the full-adoption institutions (Fig. 6 (c)). For example, the following quote provides an example showing how this challenge became more prominent when an institution moved from the non-adoption scope (i.e., no action taken) to the prep-partial scope of adoption:

We had never attempted to export and bring together different data sets for analysis, because the student record is huge and very complex and so is the VLE record. And you have to decide what you will export. And we had never tried that before. So actually, all of that turned out to be harder than we thought. It wasn’t simple and straightforward push a button and the data exported. We had to make a whole set of decisions about what to export. *U24*

The results also showed that technology enhancement (*T.En*) became more prominent among the prep-partial adoption groups (Fig. 6 (b)), compared to the none adoption group (Fig. 6 (a)), with a connection to resource challenges. For institutions that have reached full adoption, we started to see more involvement of external partnership in building the technological infrastructure (*T.ExP*), which, however, came with challenges related to ethics and privacy (*C.EaP*)( Fig. 6 (c)).

## Discussion

Based on the existing literature, several factors impact LA adoption, including stakeholder involvement, leadership, ethics, analytical capabilities, and analytical culture. The findings of this study based on ENA revealed that three of the challenges have more prominent connections with other factors – ethics and privacy, stakeholder buy-in, and resources. The “ethics and privacy” challenge was connected to multiple factors, including the ethical consideration of access and consent as well as the external stakeholder factor; the “resource” challenge was connected to factors including technology evaluation, leadership, and experts; whereas the “stakeholder buy-in” challenge was connected to various types of analytic cultures (strategic, resistant, and developing cultures). These findings show that issues around ethics and privacy, stakeholder buy-in, and resources tend to be multifaceted that require special attention, as also suggested by previous studies (Tsai et al., 2019, 2020).

Several studies illustrate that ethics plays a significant role in LA adoption (Ferguson et al., 2016; Slade & Prinsloo, 2013). However, the increasing pervasiveness of data and analytics in higher education may cause plenty of challenges (Slade & Prinsloo, 2013). The ENA results in the current study showed that both none and prep-partial adoption institutions demonstrated strong connections between seeking informed consent as an ethical factor and the challenges of ethics and privacy, which indicates that HEIs in early stages of LA adoption may particularly struggle with the ways to seek informed consent from primary stakeholders. These findings are similar to previous results reported in the literature (Tsai et al., 2021a), where the authors compared novice and experienced institutions based on years of experience with LA. They found that novice institutions had a particular challenge with data access, whereas our study provided a different angle by comparing institutions based on their scope of adoption regardless of experience. We found that both prep-partial and full adoption institutions particularly struggled with data access. Our study points to an urgent need for institutions to start their LA adoption journey by untangling issues with consent-seeking and continue to examine the access right to various types of data, e.g., the consent should be explained to the primary stakeholders about how their data will be used and who will access the data (Jones, 2019; Slade & Prinsloo, 2013).

The widespread adoption of LA can lead to positive outcomes based on a change of leadership mindset (Diaz & Fowler, 2012). Our results revealed that centralized leadership is especially prominent among the none and full adoption HEIs, whereas distributed leadership is prominent among the prep-partial adoption institutions. However, despite the leadership model, institutions in all adoption scopes are found to have connections between leadership and resource challenges, particularly the none-adoption HEIs. However, once institutions reached the implementation stages, challenges related to adoption methodologies started to emerge. Although these connections cannot suggest causality between leadership types and challenge types but rather the concurrence of both elements, the results pointed to a need for leadership awareness of different challenges institutions may face when they move into different stages (scopes) of adoption. HEIs may consider a more holistic and complex organizational leadership approach (Dawson et al., 2018; Tsai et al., 2019) that promotes adaptive leadership in response to a changing environment and demands that derive from the change.

A fully operational culture that values LA takes time to build (Rehrey et al., 2019) and can vary significantly across institutions due to their unique contexts (Hilliger et al., 2020; Kollom et al., 2021; Whitelock-Wainwright et al., 2020). The current study showed that stakeholder buy-in is the main challenge that had strong connections with the analytic culture factors. Among these connections, resistance culture is prominent among the none and prep-partial adoption HEIs, whereas strategic analytical culture (i.e., institutions had strategies to address resistance to LA) is found among the full-adoption HEIs. This finding implicates the importance of institutional strategies to increase stakeholder acceptance and ownership of LA, so as to increase the maturity and scalability of LA. For example, offering training that aims to help users understand how to use analytics tools, how to act on LA, and steps that HEIs take to protect stakeholders’ data may improve analytic culture and thus positively impact stakeholders’ buy-in (Tulasi, 2013).

Analytic capabilities are essential for collecting and analysing data and for solving complex data analytic problems. Analytic capabilities enable institutions to make data-informed decisions based on evidence (Tulasi, 2013). The existing studies draw attention to the importance of data analysis skills and capabilities in LA (Lester et al., 2017; Oster et al., 2016) and stakeholder buy-in (Ferguson et al., 2014) in LA adoption. The results of our study showed that gaps present in the understanding of and skills for LA have a strong connection with stakeholder buy-in even when institutions have reached full adoption. It also appears that the issue with capability gaps is connected to several other challenges, including resources (e.g., human resources) and ethics and privacy issues. This shows that even when institutions have reached institution-wide adoption, capability gaps can have a chain of negative impacts on an institution’s overall capacity for LA.

Stakeholder involvement and continued participation have been vital for the success of LA projects (Ferguson et al., 2014; Greller & Drachsler, 2012; Lester et al., 2017; Tsai & Gasevic, 2017; Tsai et al., 2019, 2020). Our result showed that collaboration with third parties was associated with different challenges (e.g., resources, ethics). These challenges could be related to ethical issues that occurred due to the partnership or that the institutions sought support from third parties to address challenges related to resources or ethics. The ENA findings showed that none and full adoption institutions demonstrated strong connections between external stakeholders and the resourcing challenges. This indicates evidence of engagement with external stakeholders to secure required resources for LA (e.g., technological infrastructure, software, data warehouse) despite adoption scope. In addition, there is a considerable uncertainty with regard to ethics and privacy during engagement with external stakeholders. The results of the current study showed that prep-partial adoption institutions had strong connections between external stakeholders and ethics and privacy. In other words, the prominence of ethics privacy issues out weighted resource issues in their partnership with external stakeholders. This could indicate that the HEIs that were in the early stages of adoption were paying particular attention to smoothing out ethics and privacy issues or that they had concerns related to setting protocols for data sharing with external partners. An increasing number of studies have found that there are concerns raised in HEIs about sharing of student data with external stakeholders (Selwyn, 2019; Tsai et al., 2021b). This concern should be addressed by setting a policy that safeguards stakeholders’ data from harmful use that can result from data sharing, especially in the preparation stage, as suggested above.

Sound technological infrastructure is an essential building block for LA adoption (Arnold et al., 2014b; Klein et al., 2019). Our results revealed that resources and capability are key challenges that tend to accompany the action of technology evaluation. This could mean that when institutions evaluate their IT systems, these challenges tend to surface. However, it also means that there is a high demand for resources and capability for institutions to carry out an adequate evaluation of their IT systems required for LA implementation. In particular, the capability demand seems to get more prominent when institutions move beyond the interest phase to the adoption phase (prep-partial full). This finding suggests that while resources, such as technological tools, are among the first barriers to overcome when institutions start to take an interest in LA (Macfadyen et al., 2014; Norris & Baer, 2013), institutions should be prepared to face the imminent need to scale the knowledge and skills required for LA.

## Implications

In light of the results of this research, senior managers can consider the following recommendations for adopting LA in higher education:

- Define the consent-seeking process early and continue to assess different stakeholders’ rights to access data as institutions move through different phases of adoption;
- Drive and sustain LA with adaptive leadership that can orchestrate resources including LA expertise, ensure effective adoption approaches, and bridge differences between stakeholders according to the changing needs in different phases of adoption;
- Adopt a hybrid approach when building technological infrastructure for LA to minimise the complexity of ethics and privacy issues; for instance, use in-house IT teams for general support and work with third-party for specialized projects.
- Strengthen training for critical stakeholders to scale their analytic skills and improve an appetite for using data to enhance learning and teaching.

## Conclusion

Higher education institutions (HEIs) are drawn to LA as a solution towards the enhancement of teaching and learning practice. However, several prominent challenges in the LA adoption process have impeded the scalability and effectiveness of LA. The aim of this study was to explore and untangle the connections between the challenges of LA adoption and their related factors. The study revealed three particular challenges associated with multiple adoption factors – ethics and privacy, stakeholder buy-in, and resources. This indicates that the impacts of these challenges can be at multiple levels and solutions need to be holistic considering all the associated factors. Unexpectedly, we identified a degree of similarity between none adoption and full adoption institutions in their ENA graphs, particularly with respect to challenges related to stakeholder involvement, technology, and analytic capabilities. This shows that for institutions that are still exploring possibilities to adopt LA and for those that have adopted LA throughout the institution, equal attention is needed for these three areas, even though issues may vary. The current study contributes to the body of literature about connections of challenges in LA adoption with their related factors in higher education institutions in different scopes of LA adoption. The insights obtained from the study can inform adoption strategy for institutions that are starting LA or considering expanding its scope. As interest in LA grows, it is critical that HEIs recognise the challenges that may limit the adaption of LA and address these challenges strategically and systematically.

## Limitations and future work

This study has limitations, the data used for this study was collected only from European HEIs between late 2016 and early 2017. Despite the fact that LA was a relatively new idea to many HEIs at the time the data was collected, literature (Guzmán-Valenzuela et al., 2021) continues to show that various challenges with learning analytics (LA) have stagnated the adoption rate, and the challenges discussed in this paper remain relevant today. In future study, there is a need to investigate challenges and their related factors in different parts of the world during or after Covid-19. Further, institution size and annual budget are interesting points of comparison in future work.

## Appendix 1

Table 8

**Table 8:** Independent two sample-test statistics result among the connection of the three adoption LA scope

Connections	Axises	None-Prepartial	None-Full	Prepartial-Full
Challenge and ethics	X-axis	T=-3.3069, DF=25.486, **P=**.**002808**	T=- -3.4275, DF =15.483, **P**=**0.003596**	T= -2.4987, DF=21.944, **P**=**0.02045**
	Y-axis	T=2.1377e-16 DF=18.537, P= 1	T= 5.2152e-16, DF=22.888, P=1	T= -1.3219e-16, DF=19.233, P=1
Challenge and leadership	X-axis	T= -2.2187, DF=30.401, **P=0.03412**	T=-1.8097, DF=13.427, P=0.09277	T= -2.4527, DF=11.171, **P**=**0.03178**
	Y-axis	T=5.3281e-16, DF=30.643, P=1	T=-9.8768e-17, DF=17.894, P=1	T=-1.0266e-16, DF=11.155, P=1
Challenge and analytical culture	X-axis	T= -4.8152, DF= 30.992, **P**= **3.647e-05**	T= -6.0408, DF=25.069, **P**=**2.574e-06**	T=-4.3839, DF=16.219, **P**=**0.0004487**
	Y-axis	T= 3.2123e-16, DF= 24.242, P=1	T= -5.2297e-16, DF=27.727, P=1	T= 0, DF=21.917, P=1
Challenge and analytical capability	X-axis	T= -3.5083, DF=30.98, **P=0.001402**	T= -2.6676, DF=14.082, **P=0.01831**	T= -3.1963, DF= 10.617, **P**=**0.008879**
	Y-axis	T= -1.6371e-16, DF=30.07, P=1	T= 1.0565e-16, DF=20.616, P=1	T= -3.5792e-16, DF=21.319, P=01
Challenge and stakeholder involvement	X-axis	T= -3.8018, DF=24.157, **P=0.0008607**	T= -4.5906, DF=25.751, **P=0.0001011**	T= -3.1537, DF=17.864, **P**=0.**005532**
	Y-axis	T= -1.2802e-16, DF=30.602, P=1	T= -6.3758e-16, DF= 26.628, P=1	T= 0, DF=21.947, P=1
Challenge and technology	X-axis	T=--3.3045, DF=25.644, **P**=**0.00281**	T=-3.7109, DF=28.993, **P**=**0.000872**	T= -1.6376, DF=13.423, P=0.1247
	Y-axis	T=7.6412e-17, DF=21.097, P=1	T= 5.5307e-17, DF=18.617, P=1	T=-8.0489e-17, DF=21.827, P=1

The significant difference is written in bold

## Appendix 2 Challenges and ethics

Figure 7

Figure 8

## Appendix 3:Challenges and leadership

Figure 9

Figure 10

## Appendix 4:Challenges and analytical culture

Figure 11

Figure 12

## Appendix 5: Challenges and analytical capability

Figure 13

Figure 14

## Appendix 6:Challenge and stakeholder involvement

Figure 15

Figure 16

## Appendix 7:Challenges and technology:

Figure 17

Figure 18
